# Impact of Postoperative Liver Injury on the Oncological Short- and Long-Term Outcome After Liver Resection for Hepatocellular Carcinoma

**DOI:** 10.3390/cancers18081199

**Published:** 2026-04-09

**Authors:** Katharina Lang, Oliver Beetz, Iakovos Amygdalos, Clara A. Weigle, Bengt A. Wiemann, Julian Palzer, Sebastian Cammann, Georg Wiltberger, Thomas Vogel, Florian W. R. Vondran, Franziska A. Meister, Felix Oldhafer

**Affiliations:** Department of General, Visceral, Pediatric and Transplant Surgery, University Hospital RWTH Aachen, Pauwelsstraße 30, 52074 Aachen, Germany

**Keywords:** hepatocellular carcinoma, liver resection, SAAR score, post-hepatectomy liver failure, surgical oncology, APRI ALBI

## Abstract

Background: Postoperative liver injury remains a major challenge after curative-intended liver resection for hepatocellular carcinoma (HCC), impacting both early complications and long-term survival. The recently introduced SAAR (Sum of AST/ALT Ratios) score may serve as a novel marker for predicting postoperative outcomes. Methods: This retrospective single-center study included 213 patients undergoing liver resection for HCC between January 2007 and October 2024. The SAAR score was calculated using AST/ALT ratios on postoperative days 1 and 3 and correlated with post-hepatectomy liver failure (PHLF), disease-free survival (DFS), and overall survival (OS). The predictive performance of the SAAR score was compared with that of the combined APRI + ALBI score. Results: Patients with SAAR ≥ 2 showed significantly higher rates of PHLF (OR = 2.5, *p* = 0.019) and impaired long-term outcomes. Median DFS and OS were significantly reduced in the SAAR ≥ 2 group (7 vs. 24 months, *p* < 0.001; 12 vs. 32 months, *p* = 0.004, respectively). Multivariate Cox regression confirmed SAAR ≥ 2 as an independent predictor for DFS (HR = 2.1) and OS (HR = 1.9). The APRI + ALBI score also demonstrated strong preoperative predictive value for PHLF (AUC = 0.854) but not for long-term outcome.

## Simple Summary

Liver cancer (hepatocellular carcinoma, HCC) is one of the leading causes of cancer death worldwide, and surgical removal of the tumor remains the primary curative treatment. Surgery can, however, trigger temporary liver injury whose impact on long-term prognosis is not yet fully understood. This study examined whether the SAAR score—a simple marker derived from two routine liver enzymes (AST and ALT) measured on postoperative days 1 and 3—can predict both short-term liver complications and long-term cancer outcomes in patients undergoing liver resection for HCC. In 213 patients treated at a European university hospital over 17 years, an SAAR score ≥ 2 was independently associated with significantly shorter recurrence-free and overall survival and with a higher risk of post-hepatectomy liver failure. In contrast, the preoperative APRI + ALBI score predicted liver failure but not long-term survival. These two scores appear to capture complementary aspects of risk: APRI + ALBI reflects baseline hepatic reserve, while SAAR reflects the extent of perioperative liver injury. Because SAAR relies solely on standard laboratory values, it represents a practical, low-cost tool to identify high-risk patients who may benefit from intensified follow-up or adjuvant treatment strategies after liver resection for HCC.

## 1. Introduction

Primary liver malignancies are one of the leading causes of cancer-related mortality worldwide [1]. Over the last few decades, the incidence of primary liver cancer has been increasing [2]. The majority of primary liver tumors arise from liver cells, manifesting as hepatocellular carcinoma (HCC) [3]. Among curative treatment approaches, liver resection is the mainstay option [4,5,6,7,8]. Despite continuous improvements in surgical technique and perioperative management, postoperative morbidity and mortality remain important challenges in hepatobiliary surgery [9]. Post-hepatectomy liver failure (PHLF), defined as newly occurring deterioration of synthetic, excretory, and detoxifying functions, is a major contributor to post-hepatectomy mortality [10,11,12,13,14,15,16].

Intraoperative blood loss during liver resection is associated with increased postoperative morbidity and mortality [17]. Temporary hepatic inflow occlusion by clamping the vessels within the hepatoduodenal ligament is effective in minimizing blood loss [18,19]. However, this technique induces hepatic ischemia, followed by reperfusion injury caused by a cascade of metabolic disturbances, including anaerobic metabolic processes, mitochondrial damage, oxidative stress, and release of reactive oxygen species (ROS) [20]. Transaminases, Alanine Aminotransferase (ALT) and Aspartate Transaminase (AST), are nonspecific biochemical markers for liver injury [21]. Hence, these enzymes are frequently incorporated into scoring systems to predict the outcome or to stratify the risk of patients after liver resection [22]. A well-known example is the AST to Platelet Ratio Index (APRI), which combines AST and platelet count [23]. In 2024, Lu et al. introduced the Sum of AST/ALT Ratios (SAAR), a novel predictive score to stratify patients’ long-term outcome after liver resection for HCC, measured as Disease-Free Survival (DFS) or Overall Survival (OS) [24]. This score is calculated by summing the quotients of AST and ALT on postoperative days 1 and 3, respectively. In their multicentric study, Lu et al. could show that an SAAR < 2 was a predictor of better survival, and a SAAR score between 2 and 3.5 was associated with a significantly increased risk of recurrence. Further, SAAR ≥ 3.5 correlated significantly with an elevated risk of mortality [24].

The SAAR score was originally developed in a multicenter study including patients from Asia, North America, Australia, and Europe [24]. While this approach enhances generalizability, it is inherently heterogeneous in patients’ characteristics, surgical strategies, and perioperative management. In particular, the epidemiology of underlying liver diseases differs between Western patient cohorts and Asia, with a lower prevalence of HBV and a higher proportion of alcohol and metabolic-related liver disease [25,26]. A validation in a single-center cohort allows the evaluation under standardized clinical conditions, which reduces inter-institutional variability, and thus would reinforce and further confirm the applicability of the score [25,26].

The aim of our study was to evaluate the SAAR score in a large, European single-center cohort of 213 curative-intent resections for HCC. In addition, due to the usefulness of transaminases as a marker for liver damage, our study aimed to evaluate the prognostic value of the SAAR score for post-hepatectomy liver failure (PHLF) after liver resection and to compare the SAAR with the recently introduced APRI + ALBI score, which has been reported to outperform functional tests such as LiMAx [27].

## 2. Methods

### 2.1. Study Design

The retrospective study was conducted using patient data obtained from a prospectively maintained institutional database, as described before [28]. A total of 272 Patients who received a curative-intended liver resection for HCC between January 2007 and October 2024 at RWTH Aachen University Hospital were included in the study. Surgical procedures were performed in a standardized manner, either minimally invasive or open. Vascular inflow occlusion (Pringle maneuver) was applied selectively but not as a routine measure. Patients with missing laboratory values of AST or ALT on postoperative days 1 or 3 were excluded from analysis (n = 59). The last date of follow-up was 12 February 2025, resulting in a patient follow-up time of at least five months.

The study adhered to the principles of the Declaration of Helsinki and ICH-GCP guidelines. Approval was obtained from the RWTH Aachen University IRB (EK 115/20; EK 341/21). Owing to the retrospective design and use of routine clinical data, informed consent was waived.

### 2.2. Definition of Variables

Postoperative complications were recorded using the Clavien-Dindo classification and the Comprehensive Complication Index (CCI) [29,30]. The period of postoperative hospital stay was considered, as well as probable further hospital stays in case of rehospitalization within 14 days after discharge. According to the previously published formula, the Sum of AST/ALT Ratios (SAAR) was calculated as $SAAR=\;\frac{1}{\sqrt{2}}(\frac{AST1}{ALT1}+\frac{AST3}{ALT3})$ [24]. AST and ALT were determined on the first and third day postoperatively. Patients were divided into two groups: SAAR < 2 and SAAR ≥ 2. Patients with a SAAR-score ≥ 2 were classified as having an increased risk for recurrence or premature death. The SAAR cut-off value ≥ 2 was prespecified according to the original publication, which proposed two thresholds (≥2 and ≥3.5) [24]. The lower threshold was selected as it allows the identification of a larger proportion of patients at increased risk. APRI was calculated as APRI = $\frac{\frac{AST\;(\frac{U}{L})}{AST\;Upper\;Limit\;of\;Normal\;(U/L)}\times 100}{Platelet\;Count\;(10^{9}/L)}$ as previously published [23]. To determine the ALBI Score, preoperative albumin and bilirubin values were analyzed using the following formula: ALBI=(log10 bilirubin (μmol/L)× 0.66)+(albumin gL× −0.085) [31]. Based on established methods, the combined APRI + ALBI score was derived [32]. A resection of more than three segments was defined as a major resection.

Post-hepatectomy liver failure (PHLF) was defined according to the International Study Group of Liver Surgery (ISGLS) as Grade B + C [33]. These included all patients with abnormal laboratory values who required additional clinical treatment with (Grade C) or without (Grade B) the invasive treatment.

### 2.3. Statistical Analysis

Continuous data were presented as median [interquartile range], categorical data as absolute numbers (relative frequencies). For categorical data, groups were compared using either the Chi-square or Fisher’s exact test, as appropriate. After assessing the normality of the data distribution using the Kolmogorov–Smirnov test, metric data were compared using either Student’s *t*-test or the Mann–Whitney U test, depending on the distribution. Endpoints were set as DFS and OS, defined as the time interval between operation and recurrence or death, or last follow-up in patients without these events. Patients who died during the initial hospital stay were excluded from the long-term outcome. For survival analyses, Kaplan–Meier curves were generated, and differences were assessed employing the log-rank test. Uni and multivariate Cox regressions were performed. Receiver Operating Characteristics (ROC) curves were constructed, and the Area Under the Curve (AUC) was calculated to quantify the accuracy of the predictive model. A two-sided *p*-value of < 0.05 was regarded as significant. To explore the potential interaction between SAAR and APRI + ALBI, an interaction term was included in a Cox regression model.

To assess potential selection bias, baseline characteristics of included and excluded patients were compared. Continuous variables were analyzed using the Mann–Whitney U test, categorical variables using the Chi-square test or Fisher’s as appropriate.

All statistical analyses were performed using SPSS (IBM Corp., Released 2022. IBM SPSS Statistics for Macintosh, Version 29.0, Armonk, NY, USA: IBM Corp.).

## 3. Results

### 3.1. Baseline Characteristics

Of the initial *n* = 272 patients, *n* = 59 were excluded due to missing laboratory values, resulting in *n* = 213 patients for the final analysis. Baseline characteristics of included and excluded patients are summarized in Supplementary Table S1. Most variables were comparable between the two groups. Tumor size was significantly larger in excluded patients. The following analyses were performed in the group of the included patients. The median age was 71 [62–76] years, and 161 patients (75.6%) were male. The median BMI measured 26.8 [23.7–30.5] kg/m^2^. Underlying liver cirrhosis was present in *n* = 105 (49.3%) cases. The median tumor size reached 46.0 [30.0–70.0] mm, and 31.0% (*n* = 66) suffered from multiple tumor nodes. A major resection (>3 segments) was performed in *n* = 66 (31.0%) cases. The median SAAR-value was 1.4 [1.2–1.9], resulting in *n* = 171 patients with a SAAR score smaller than 2 and *n* = 42 with a SAAR score larger than or equal to 2. Significant differences between the two groups were observed in gender, underlying cirrhosis, tumor size, and Model for End-Stage Liver Disease score (MELD). Otherwise, they were comparable in terms of baseline characteristics. The details are shown in Table 1 and Table 2.

### 3.2. Impact of the SAAR Score on PHLF

**Figure 1:**
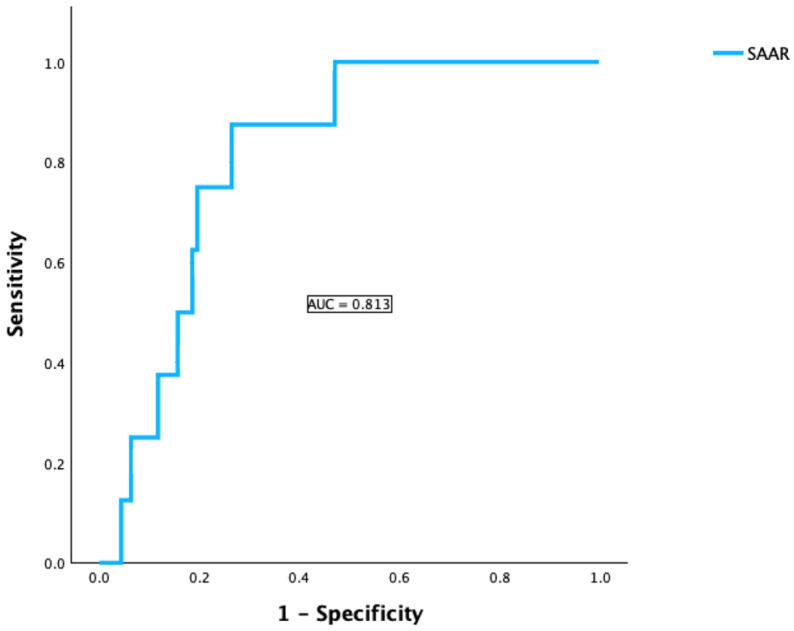
ROC and AUC for the prediction potential of SAAR for the occurrence of PHLF.

**Figure 2:**
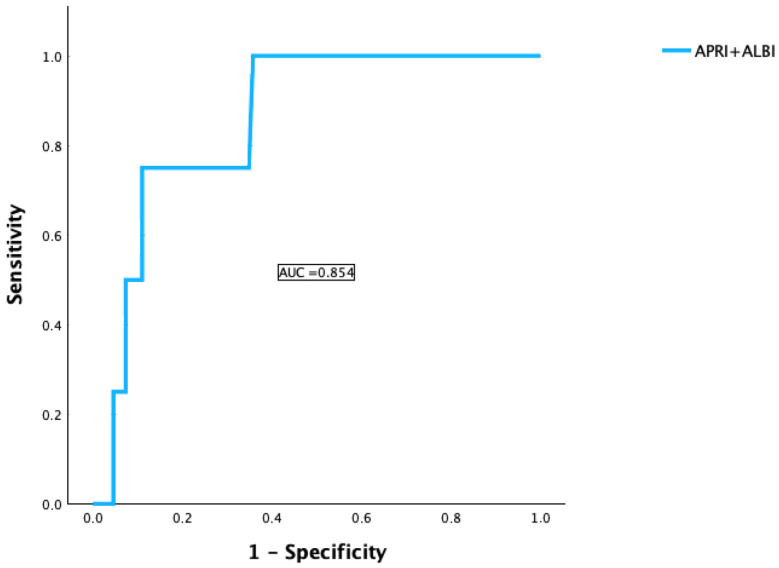
ROC and AUC for the prediction potential of APRI + ALBI for the occurrence of PHLF.

In our cohort using ISGLS criteria, 75.6% showed no PHLF, 20.2% had grade A, 1.4% had grade B, and 2.3% suffered from grade C. The distribution was similar between patients with SAAR < 2 and SAAR ≥ 2, although grade C PHLF was more frequent in the higher SAAR group (7.1% vs. 1.2%, *p* = 0.049, Table 1). To investigate a possible association between higher SAAR scores and more severe postoperative liver failure, we analyzed whether SAAR serves as a predictive factor for the development of PHLF. The logistic regression showed that the SAAR value was a significant positive predictor of PHLF with an Odds Ratio (*OR*) = 2.5 (95% CI: 1.2–5.3) and *p* = 0.019. ROC analysis was performed to assess predictive performance. The AUC of 0.813 (95% CI: 0.715–0.910) indicated good discriminatory ability (Figure 1). In addition, we evaluated the prognostic potential of the combined APRI + ALBI score to determine its added value in risk stratification. APRI + ALBI was a significant predictor of PHLF with an *OR* = 2.5 (95% CI: 1.1–5.7, *p* = 0.031). AUC = 0.854 (95% CI: 0.724–0.985, *p* < 0.001) indicated good discriminatory ability (Figure 2).

### 3.3. Impact of SAAR-Value on DFS and OS

**Figure 3:**
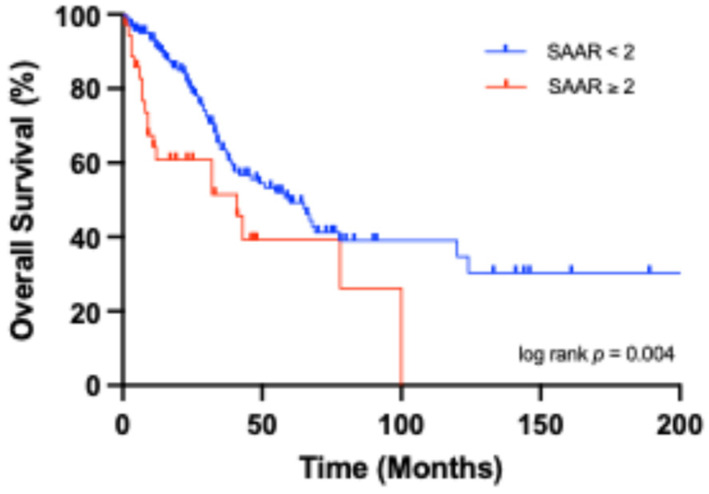
Kaplan–Meier curve showing OS stratified by SAAR ≥ 2.

**Figure 4:**
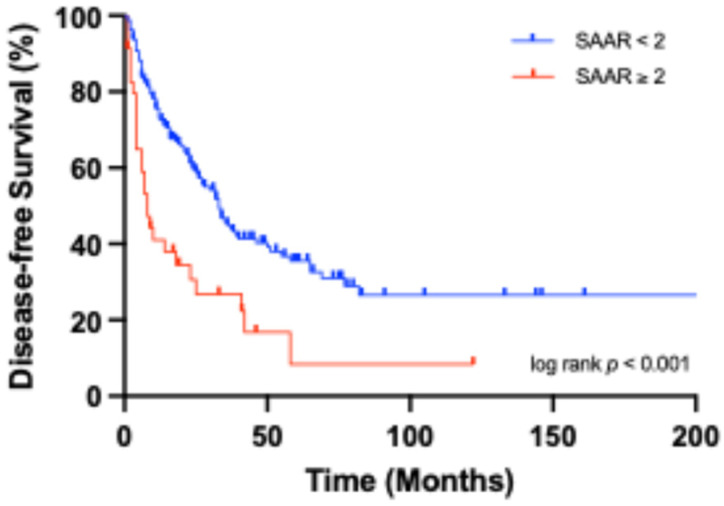
Kaplan–Meier curve showing DFS stratified by SAAR ≥ 2.

Median DFS for the entire cohort was 21.0 [7.0–40.0] months. Patients with a SAAR score < 2 had a significantly longer DFS of 24.0 [10.0–45.0] months compared to those with a SAAR score ≥ 2, who had a median DFS of 7.0 [2.5–21.0] months. Median OS was 31.0 [14.0–49.0] months for all patients, with the SAAR < 2 group showing a longer OS of 32.0 [18.0–56.0] months versus 12.0 [5.5–37.0] months in the SAAR ≥ 2 group. Kaplan–Meier curves showed highly significant (*p* < 0.001) reduced DFS in patients with SAAR ≥ 2 compared to those with SAAR < 2 (Figure 3). Further Kaplan–Meier analyses showed significant differences between the groups in OS (*p* = 0.004, Figure 4). Consistent with these findings, the Hazard Ratio (HR) for OS resulted in 2.1 (95% CI: 1.3–3.5, *p* = 0.003, Table 3), revealing a significantly increased risk of premature death. In contrast, APRI + ALBI was not significantly associated with either outcome. For DFS, the HR was 1.4 (95% CI: 0.9–2.3, *p* = 0.147, Table 4) and for OS it was 1.4 (95% CI: 0.8–2.7, *p* = 0.214, Table 3). In a supplementary analysis, SAAR was evaluated as a continuous variable in a Cox regression model and remained significantly associated with DFS (HR = 1.9, 95% CI: 1.4–2.4, *p* < 0.001) and OS (HR = 1.7, 95% CI: 1.3–2.2, *p* < 0.001). In a Cox regression model including an interaction term between SAAR and APRI + ALBI low-risk category, no statistically significant interaction was observed (*p* = 0.943).

In addition, univariate analysis revealed that the SAAR ≥ 2 was associated with a 2.3 times higher risk of developing recurrence (Hazard Ratio (HR) = 2.3 (95% CI: 1.5–3.6, *p* < 0.001; Table 4). Several additional factors were tested in univariate analysis for their association with both outcomes, DFS and OS (Table 3 and Table 4). Hence, all significant factors were included in a multivariate Cox regression to assess the combined effect while adjusting for potential confounders. The multivariate model for DFS showed that SAAR ≥ 2 remained an independent predictor for DFS with HR = 2.1 (95% CI: 1.3–3.3, *p* = 0.001, Table 4). Further UICC Stage ≥ III and cirrhosis likewise emerge as independent predictors for the outcome, with HR = 3.3 (95% CI: 2.1–5.2) and 1.9 (95% CI: 1.3–2.9), respectively, and *p* < 0.001 (Table 4) each. In contrast, R1 resection and the presence of multiple lesions lost their significance (HR = 1.3 (95% CI: 0.8–2.3), *p* = 0.290, HR = 1.4 (95% CI: 1.0–2.1), *p* = 0.069, Table 4).

Likewise, in the multivariate analysis, SAAR ≥ 2 persisted as an independent predictor for OS with HR = 1.9 (95% CI: 1.1–3.2, *p* = 0.021, Table 3). Again, UICC stage ≥ III and cirrhosis also remained independent predictors for the outcome (HR = 3.7 (95% CI: 2.2–6.4), *p* < 0.001, HR = 1.7 (95% CI: 1.0–2.7), *p* = 0.034, Table 3) supplemented by R1 resection (HR = 2.0 (95% CI: 1.1–3.6), *p* = 0.019, Table 3). The presence of multiple lesions did not reach significance with HR = 1.2 (95% CI: 0.8–1.9, *p* = 0.381, Table 3).

## 4. Discussion

Despite continuous improvement in therapy, HCC remains the third leading cause of cancer-related deaths worldwide [1]. PHLF and early tumor recurrence continue to represent major determinants of poor prognosis following curative liver resection for HCC [16,34]. Among the contributing factors, peri- and postoperative liver injury has emerged as a potentially modifiable element that may influence both short- and long-term outcomes.

The present study demonstrated the prognostic value of transaminases and the recently developed SAAR score for short- and long-term outcomes following curative liver resection in patients with HCC in a European cohort. Elevated transaminases are known for being markers of liver injury [35]. Liver damage may impair not only postoperative regeneration but also oncological outcome. Postoperatively elevated transaminases are assumed to be risk factors for early recurrence and higher tumor aggressiveness in HCC [36,37]. In previous findings, Lu et al. (2024) reported a significant association between an elevated postoperative SAAR score between 2 and 3.5 and a decreased DFS and between SAAR ≥ 3.5 and a reduced OS [24]. Our results not only confirm these associations with DFS, but even suggested that already SAAR values ≥ 2 are significantly correlated with poorer OS. Thus, our findings highlight the relevance of lower SAAR cut-off values as prognostic indicators for both oncological and overall outcomes. The adoption of a unified threshold (SAAR ≥ 2) could enhance clinical applicability. Although applying a binary cut-off may theoretically result in a loss of information, SAAR remained significantly associated with DFS and OS when analyzed as a continuous variable, confirming the robustness of this association. In this context, it must be noted that although SAAR appeared to lose prognostic relevance within the APRI + ALBI low-risk subgroup in a supplementary analysis, the absence of a statistically significant interaction effect suggests that this observation should be interpreted with caution and regarded as hypothesis-generating rather than conclusive.

In contrast to novel parameters such as circulating tumor DNA, the SAAR score is easily obtainable and non-invasive, as it relies on routinely available laboratory values. Its assessment in the immediate postoperative phase makes it a potential tool for early stratification of patients’ risk for premature recurrence and mortality. Consequently, the SAAR score may inform individualized postoperative management planning. Patients with elevated SAAR values could be specifically identified for intensified follow-up treatment or consideration of adjuvant therapies, particularly in the current era of immunotherapy, as shown by the promising results of the IMbrave050 trial [38].

In addition, we were able to show a significant prognostic relevance of the SAAR score on the development of PHLF. Thus, calculating the SAAR value provides a non-invasive tool for short-term risk stratification and therapeutic planning. However, a major limitation of this marker is that it can only be assessed in the postoperative setting, which restricts its usability for preoperative decision-making. To address this, we further included the combined APRI + ALBI score in our analysis [32]. This composite score has been shown to outperform other established scores, such as Indocyanine Green Clearance, LiMAx, or Child Pugh, in forecasting PHLF [27,39,40]. In line with these findings, we found APRI + ALBI to be a significant preoperative predictor of PHLF in our HCC cohort.

Our results, demonstrating a significant association for both the SAAR as well as the APRI + ALBI score with PHLF, confirm their role as important tools for risk prediction in patients after curative-intended liver resection for HCC. While the APRI + ALBI scores are well-established predictors of short-term outcomes following liver resection, evidence regarding their impact on long-term oncological outcomes, such as DFS and OS, remains limited. Consistent with this, our study found no significant association between APRI/ALBI scores and DFS or OS. Not surprisingly, local inflammation may carry greater prognostic weight than preoperative liver function. On the other hand, when we repeated the Kaplan–Meier analyses for DFS and OS exclusively in patients with an APRI + ALBI score of ≤−2.46, classified as the low-risk group, the SAAR score no longer showed prognostic relevance. Patients with preserved hepatic function might be able to compensate peri- or postoperative injury more effectively. However, a Cox regression model testing the interaction of SAAR and APRI + ALBI low-risk category did not demonstrate a significant interaction. In the present study, APRI + ALBI and SAAR appeared to capture different aspects of perioperative risk and should be considered complementary rather than interchangeable markers. APRI + ALBI mainly reflects preoperative hepatic functional reserve [32] and was associated with the occurrence of PHLF; however showed no significant relationship with long-term oncological outcome. In contrast, SAAR reflects hepatic ischemia and reperfusion injury postoperatively [41] and was associated with both short- and long-term outcomes. Taken together, these findings suggest that the prognostic impact of postoperative liver injury may depend on baseline liver function and that SAAR provides prognostic information beyond hepatic reserve alone. There are various pathways that might contribute, i.e., hepatic-ischemia–reperfusion injury may promote tumor recurrence through increased proliferation, angiogenic factors, and implantation of circulating cancer cells [42,43]. In addition, reperfusion injury may trigger an inflammatory response and impair liver regeneration, leading to an unfavorable microenvironment promoting tumor recurrence [44]. The precise biological mechanisms reflected by SAAR remain to be clarified. Future studies correlating SAAR with histopathological markers of hepatic injury and inflammation (e.g., neutrophil-to-lymphocyte ratio in the specimen or specific cytokine profiles) may help to better understand the underlying processes captured by this marker.

The present study is subject to limitations, most notably its retrospective nature and single-center design, which may limit the generalizability of the results to a broader patient population. A major limitation is the exclusion of 59 patients due to missing laboratory values required for SAAR calculation. Although baseline characteristics were mostly comparable apart from tumor size, a possible selection bias should be considered when interpreting the results. Further, although the overall cohort size was adequate for primary analyses, the distribution of patients between SAAR subgroups was unequal. The small number of patients in the high-risk group (*n* = 42) may have limited the statistical power of subgroup analyses, especially relevant for infrequent outcomes such as grade C PHLF. These findings have to be interpreted with caution, given the limited sample size.

In conclusion, the SAAR value shows potential as a predictive marker for both short-term and long-term outcomes after curative liver resection in patients with HCC. As the score is calculated postoperatively, its utility for preoperative decision-making is inherently limited. Nevertheless, patients with higher SAAR scores may benefit from intensified surveillance strategies, including closer and more frequent follow-up assessments to facilitate early detection of potential recurrence. Furthermore, the SAAR score may assist in identifying patients at increased risk who could be considered for enrollment in clinical trials evaluating adjuvant therapies or for individualized postoperative management approaches, particularly in the context of emerging adjuvant treatment strategies. Due to its limitation of postoperative calculation, the combination with the preoperative APRI + ALBI score allows a more comprehensive assessment of perioperative risk. Since SAAR is based on routinely available laboratory parameters, it may represent a simple and cost-effective tool for postoperative risk stratification in clinical practice. To understand underlying processes, further investigations on the correlation of SAAR with histopathological markers of inflammation will be required. Further studies with larger numbers of patients are needed to verify and comprehensively evaluate our findings.

## 5. Conclusions

In the interim, SAAR can be easily calculated from routine postoperative labs and may serve as a practical tool to identify patients who warrant intensified surveillance or consideration for adjuvant therapy protocols.

## Figures and Tables

**Table 1:** Baseline demographic and clinical characteristics by SAAR groups.

Variable	All Cases	SAAR < 2 Subgroup	SAAR ≥ 2 Subgroup		
	N_abs_ (N_%_)/Median [IQR]	N_abs_ (N_%_)/Median [IQR]	N_abs_ (N_%_)/Median [IQR]	Missing Value (%)	*p*-Value
Number	213 (100)	171 (80.3)	42 (19.7)		
Male gender	161 (75.6)	136 (79.5)	25 (59.5)	0	0.007
Age	71.0 [62.0–76.0]	71.0 [62.0–76.0]	70.0 [62.8–74.3]	0	0.618
Body weight (kg)	80.0 [70.0–90.0]	80.0 [70.0–92.0]	80.0 [66.5–89.3]	0	0.361
Body height(cm)	173.0 [168.0–178.0]	174.0 [168.0–180.0]	172.0 [164.5–175.0]	0	0.039
BMI (kg/m^2^)	26.8 [23.7–30.5]	26.8 [23.8–30.1]	25.8 [23.1–31.9]	0	0.797
ASA Score				0	0.283
<3	76 (35.7)	64 (37.4)	12 (28.6)		
≥3	137 (64.3)	107 (62.5)	30 (71.4)		
Underlying disease					
HBV-infection	26 (12.2)	22 (12.9)	4 (9.5)	104 (48.8)	0.112
HCV-infection	25 (11.7)	20 (11.7)	5 (11.9)	104 (48.8)	0.337
C2	26 (12.2)	18 (10.5)	8 (19)	104 (48.8)	0.671
Cirrhosis	108 (50.7)	76 (44.4)	32 (76.2)	0	<0.001
AFP preOP (μg/mL)	6.250 [2.7–97.0]	6.0 [2.1–101.5]	12.9 [5.1–54.3]	133 (62.4)	0.168
Tumor size (mm)	46.00 [30.0–70.0]	50.0 [35.0–74.8]	30.0 [25.0–43.0]	4 (1.9)	<0.001
Number of tumor nodes	1.0 [1.0–2.0]	1.0 [23.8–30.1]	1.0 [1.0–3.0]	4 (1.9)	0.056
Multiple lesions	66 (31.0)	49 (28.7)	17 (40.5)	4 (1.9)	0.129
Macrovascular Invasion	21 (9.9)	16 (9.4)	5 (11.9)	7 (3.3)	0.576
Hepatectomy				2 (0.9)	0.056
Major (≥3 segments)	66 (31.0)	58 (33.9)	8 (19.0)		
Minor (<3 segments)	145 (68.1)	111 (64.9)	34 (81.0)		
Margin				2 (0.9)	0.231
R0	188 (88.3)	153 (89.5)	35 (83.3)		
R1	9 (4.2)	7 (0.04)	3 (7.1)		
R2	5 (2.3)	4 (2.3)	1 (2.4)		
Rx	9 (4.2)	5 (2.9)	4 (9.5)		
UICC-Stage				5 (2.3)	0.506
I/II	171 (80.3)	135 (79.0)	36 (85.7)		
III/IV	37 (17.4)	31(18.1)	6 (14.3)		
BCLC				7 (3.3)	0.115
0	11 (5.2)	8 (4.7)	3 (7.1)		
A	122 (57.3)	105 (61.4)	17 (40.5)		
B	54 (25.4)	39 (22.8)	15 (35.7)		
C	19 (8.9)	15 (8.8)	4 (9.5)		
MELD	7.0 [7.0–9.0]	7.0 [6.8–9.0]	9.0 [7.0–11.0]	2 (0.9)	<0.001

**Table 2:** Treatment outcomes by SAAR groups.

Variable	All Cases	SAAR < 2 Subgroup	SAAR ≥ 2 Subgroup		
	N_abs_ (N_%_)/Median [IQR]	N_abs_ (N_%_)/Median [IQR]	N_abs_ (N_%_)/Median [IQR]	Missing Value (%)	*p*-Value
Clavien-Dindo				0	0.386
<3b	186 (87.3)	151 (88.3)	35 (83.3)		
≥3b	27 (12.7)	20 (11.7)	7 (16.7)		
CCI	15.0 [0.0–30.8]	25.0 [0.0–30.8]	16.2 [8.7–42.5]	0	0.231
Days in Hospital	8.0 [5.0–14.0]	7.0 [5.0–14.0]	8.0 [4.75–13.25]	0	0.801
Days in ICU	2 [1.0–1.0]	[1.0–1.0]	[0.0–1.3]	0	0.923
ISGLS PHH				0	0.062
no	188 (88.3)	154 (90.1)	34 (81)		
A	17 (8.0)	13 (7.6)	4 (9.5)		
B	1 (0.5)	0 (0)	1 (2.4)		
C	7 (3.3)	4 (2.3)	3 (7.1)		
ISGLS PHLF				1 (0.5)	0.075
no	161 (75.6)	129 (75.4)	32 (76.2)		
A	43 (20.2)	37 (21.6)	6 (14.3)		
B	3 (1.4)	2 (1.2)	1 (2.4)		
C	5 (2.3)	2 (1.2)	3 (7.1)		
ISGLS Bile Leakage				0	0.501
no	191 (89.7)	150 (87.7)	41 (97.6)		
A	5 (2.3)	5 (2.9)	0 (0)		
B	12 (5.6)	11 (6.4)	1 (2.4)		
C	5 (2.3)	5 (2.9)	0 (0)		
In-hospital mortality	13 (6.1)	8 (4.7)	5 (11.9)	0	0.080

**Table 3:** Results of uni- and multivariate analyses for OS.

Variable	HR (95% CI)	*p* Value	Adjusted HR (95% CI)	*p* Value
SAAR ≥ 2	2.129 (1.301–3.482)	0.003	1.880 (1.101–3.209)	0.021
Age ≥ 60 years	1.806 (0.961–3.395)	0.066		
ASA ≥ 3	1.545 (0.995–2.399)	0.053		
UICC Stage III/IV	3.551 (2.057–5.459)	<0.001	3.709 (2.166–6.352)	<0.001
R1 resection	2.891 (1.650–5.064)	<0.001	2.000 (1.120–3.571)	0.019
Cirrhosis	1.818 (1.183–2.795)	0.006	1.681 (1.041–2.716)	0.034
Multiple resection	1.906 (1.244–2.921)	0.003	1.224 (0.779–1.922)	0.381
Major resection	1.264 (0.822–1.943)	0.285		
APRI + ALBI	1.421 (0.816–2.7474	0.214		

**Table 4:** Results of uni- and multivariate analyses for DFS.

Variable	HR (95% CI)	*p*-Value	Adjusted HR (95% CI)	*p*-Value
SAAR ≥ 2	2.329 (1.522–3.566)	<0.001	2.094 (1.341–3.270)	0.001
Age ≥ 60 years	1.359 (0.813–2.269)	0.242		
ASA ≥ 3	1.451 (0.990–2.126)	0.056		
UICC Stage III/IV	2.993 (1.974–4.538)	<0.001	3.336 (2.123–5.242)	<0.001
R1 resection	1.987 (1.188–3.325)	0.009	1.329 (0.785–2.252)	0.290
Cirrhosis	2.134 (1.478–3.083)	<0.001	1.972 (1.325–2.933)	<0.001
Multiple lesions	2.059 (1.426–2.973)	<0.001	1.424 (0.973–2.085)	0.069
Major resection	1.071 (0.728–1.575)	0.727		
APRI ALBI	1.423 (0.883–2.293)	0.147		

## Data Availability

All relevant data were reported within the manuscript and the Supplementary Files. Further supporting data will be provided upon written request addressed to the corresponding author.

## Supplementary Materials

The following supporting information can be downloaded at: https://www.mdpi.com/article/10.3390/cancers18081199/s1, Table S1. Comparison of Baseline Characteristics between included and excluded patients.
