# Supplementary material for: Impact of Postoperative Liver Injury on the Oncological Short- and Long-Term Outcome After Liver Resection for Hepatocellular Carcinoma
Source: Cancers (Basel). 2026 Apr 9;18(8):1199. doi: 10.3390/cancers18081199 (PMC13114595; doi:10.3390/cancers18081199)
Supplement: Supplementary file 1 [file cancers-18-01199-s001.zip › cancers-4158925-supplementary.pdf]

Supplementary Table 1. Comparison of Baseline Characteristics between included and excluded patients.

**Variable**	**Included (*n* = 213)**	**Excluded (*n* = 59)**		
	N_{abs} ($N_{\%}$) / *Median [IQR]*	N_{abs} ($N_{\%}$) / *Median [IQR]*	Missing Value (%)	*p*
Age	71.0 [62.0-76.0]	67.0 [59.0-75.0]	0	0.059
Male gender	161 (75.6)	42 (71.2)	0	0.492
Underlying disease				
HBV-infection	26 (12.2)	4 (6.8)	135 (49.6)	0.275
HCV-infection	25 (11.7)	7 (11.9)	135 (49.6)	0.818
C2	26 (12.2)	9 (15.3)	135 (49.6)	0.370
Cirrhosis	108 (50.7)	30 (50.8)	0	0.984
MELD	7.0 [7.0-9.0]	7.0 [7.0-9.0]	2 (0.7)	0.790
Tumor Size (mm)	46.00 [30.0-70.0]	56.0 [36.0-85.0]	4 (1.5)	**0.024**
Hepatectomy			2 (0.7)	0.266
Major (≥ 3 segments)	66 (31.0)	23 (39.0)		
Minor (< 3 segments)	145 (68.1)	36 (61.0)		
